# Exostosis

**Published:** 1867-11

**Authors:** 


### "EXOSTOSIS."

By the above term we mean a hypertrophy, or increased growth of the cementum. Many cases of facial neuralgia and toothache are due to enlargements of the roots of the teeth. A deposit of cementum, abnormal in quantity, causes pressure on the dental periosteum, unless it takes place so gradually that room is made for it by absorption of the walls of the socket.

Usually the first manifestations of pain are slight. The patients are apt to speak of "grumbling" pains. The suffering is not often continuous, but comes on in paroxysms, caused probably, by local or general disturbance of the circulation. In malarious districts the paroxysms are often strictly periodical; and the disease is, hence, often called "ague of the face," "dumb ague," etc. And, to accord with these names, the treatment often resorted to is strictly of the anti-periodic, or *anti-malarial* character. Quinine, bark, iron, arsenious acid, strychnine, and kindred agents are given, and sometimes with decided relief for a time. The malarial poison, by disturbing the circulation, produces a determination of blood to, if not congestion or inflammation in the dental periosteum, thus causing sufficient pressure to produce pain; and when the febrile reaction is over, the pressure is relieved, and the pain abates. It is on this principle that anti-malarial treatment gives relief.

A case of cemental exostosis may progress for months and years, the patient suffering, more or less intensely, in paroxysms, often unable to locate the origin of the trouble, and finally terminate in complete relief, without the removal of the offending organ. Years afterward it may become advisable to extract the tooth—as in preparing the mouth for artificial substitutes—when the excess of cementum explains all the past suffering. That many spontaneous cures are thus observed by all operators, there can be no doubt. Indeed, in many cases, the patients affirm that they have at no time felt pain. On being closely questioned, when an exostosed root has been extracted, they will sometimes admit that there had been at times a little "grumbling," or burning, or an "itching sensation," but so long ago they had almost forgotten the fact.

"Exostosis" is not a favorite disease with Dental practitioners; nor is it very popular with patients. Sound teeth are often extracted on account of it, the patient becoming so desperate that if the family Dentist will not extract according to order, another operator is resorted to; and, unfortunately, in the present state of practice, patients have but little difficulty in obtaining any dental mutilation, however outrageous it may be. Often too, as a dernier resort, respectable practitioners extract with the hope of giving relief; and if the extraction reveals a case of hypertrophied roots, he congratulates himself on having done a capital thing, and triumphantly exhibits the offending member to the patient, who goes away light-hearted, light-nerved, while but a trifle lighter in pocket.

Not many years ago it was a common sentiment in the profession that if a tooth ached, extraction was indicated; later still, if alveolar abscess developed, extraction was considered the appropriate treatment. These sentiments are not now held by any in the profession. But a large majority of our professional acquaintances speak and act as if "exostosis" were not amenable to treatment. When they have extracted a tooth thus diseased, they appear to think they have done all that our science is capable of doing for the case.

But do not the spontaneous cures referred to above, and the many cases which have never given pain, suggest something entirely different?

In some constitutions the blood appears to contain an excess of bone-making material. Such are liable to this disease. Any thing producing frequent, or prolonged, but not severe irritation of the periosteum, is likely to cause an ossific deposit in the part irritated. Hence, teeth which have to sustain more than the normal amount of pressure in mastication, are especially liable to "exostosis." It is on this principle that the disease is so frequently found in isolated teeth, and those which do not antagonize *directly* with their opponents. On the same principle it is often found on the roots of teeth which have lost their pulps. The periosteum takes on increased circulation to preserve the vitality of the tooth; and this often results in increased bony deposit.

In the *treatment* of "exostosis" two things are indicated: one is to arrest the deposit; the other to excite absorption so as to make room for the matter already deposited. Palliative treatment, for the comfort of the patient is desirable. It is scarcely necessary here to go into detail.

Fortunately both the indications above mentioned may be met by one and the same medicine; and that is the well known Iodide of Potassium. It may be given at the rate of half a dram to a dram each day. Sometimes it should be continued a week or two without interval, when it may be suspended, to be resumed in a few days if the symptoms demand it.

For the *modus operandi* of this medicine, the reader is referred to the last number of Vol. XX, of the REGISTER, or to WATT'S CHEMICAL ESSAYS, published by Dr. S. S. WHITE. W.

